# Supplementary material for: Cost-utility analysis of primary HPV testing through home-based self-sampling in comparison to visual inspection using acetic acid for cervical cancer screening in East district, Sikkim, India, 2023
Source: PLoS One. 2024 Aug 13;19(8):e0300556. doi: 10.1371/journal.pone.0300556 (PMC11321578; doi:10.1371/journal.pone.0300556)
Supplement: S1 Table — (DOCX) [file pone.0300556.s001.docx]

**S1 Table: EQ-5D-5L frequencies and proportions reported by dimensions and level**

|  |  | **Response**  **n (%)** | |
| --- | --- | --- | --- |
| **EQ-5D-5L Dimension** | **Category** | **VIA (N=95)** | **HPV (N=95)** |
| **Day 1** | | | |
| Mobility | No problem | 95 (100) | 95 (100) |
|  | Some problem | 0 | 0 |
| Self-care | No problem | 95 (100) | 95 (100) |
|  | Some problem | 0 | 0 |
| Usual activities | No problem | 95 (100) | 95 (100) |
|  | Some problem | 0 | 0 |
| Pain/discomfort | No problem | 79 (83) | 95 (100) |
|  | Some problem | 16 (17) | 0 |
| Anxiety/Depression | No problem | 89 (93) | 95 (100) |
|  | Some problem | 6 (7) | 0 |
| **Day 8** | | | |
| Mobility | No problem | 91 (100) | 93 (100) |
|  | Some problem | 0 | 0 |
| Self-care | No problem | 91 (100) | 93 (100) |
|  | Some problem | 0 | 0 |
| Usual activities | No problem | 91 (100) | 93 (100) |
|  | Some problem | 0 | 0 |
| Pain/discomfort | No problem | 91 (100) | 93 (100) |
|  | Some problem | 0 | 0 |
| Anxiety/Depression | No problem | 86 (94.5) | 93 (100) |
|  | Some problem | 5 (5.5) | 0 |
