# Supplementary material for: Cost-utility analysis of primary HPV testing through home-based self-sampling in comparison to visual inspection using acetic acid for cervical cancer screening in East district, Sikkim, India, 2023
Source: PLoS One. 2024 Aug 13;19(8):e0300556. doi: 10.1371/journal.pone.0300556 (PMC11321578; doi:10.1371/journal.pone.0300556)
Supplement: S2 Table — (DOCX) [file pone.0300556.s002.docx]

**S2 Table: Comparison of ICER and unit cost for different cervical cancer screening scenarios**

|  | **ICER** | **Incremental cost** | **Unit cost of VIA (Rs)** | **Unit cost of HPV** |
| --- | --- | --- | --- | --- |
| Current scenario | -14,459 | -326 | 1,597 | 1,271 |
| Both VIA & HPV in urban area | -53,538 | -1209 | 2479 | 1271 |
| Both VIA & HPV in rural area | -21,372 | -482 | 1,597 | 1115 |
| Consumable priced for mainland | -884 | -20 | 1291 | 1271 |
| Reduction in cost of HPV kits |  |  |  |  |
| 2% reduction | -14867 | -336 | 1,597 | 1262 |
| 5% reduction | -15480 | 349 | 1,597 | 1248 |
| 10% reduction | -16501 | -373 | 1,597 | 1225 |
| Clinician sampling of HPV at facility | -11236 | -254 | 1,597 | 1344 |
| Self-sampling at facility | -14290 | -323 | 1,597 | 1275 |
