# Supplementary figures and images for: Cost-utility analysis of primary HPV testing through home-based self-sampling in comparison to visual inspection using acetic acid for cervical cancer screening in East district, Sikkim, India, 2023
Source: PLoS One. 2024 Aug 13;19(8):e0300556. doi: 10.1371/journal.pone.0300556 (PMC11321578; doi:10.1371/journal.pone.0300556)

**S1 Fig: Flow of screening process in HPV and VIA group**


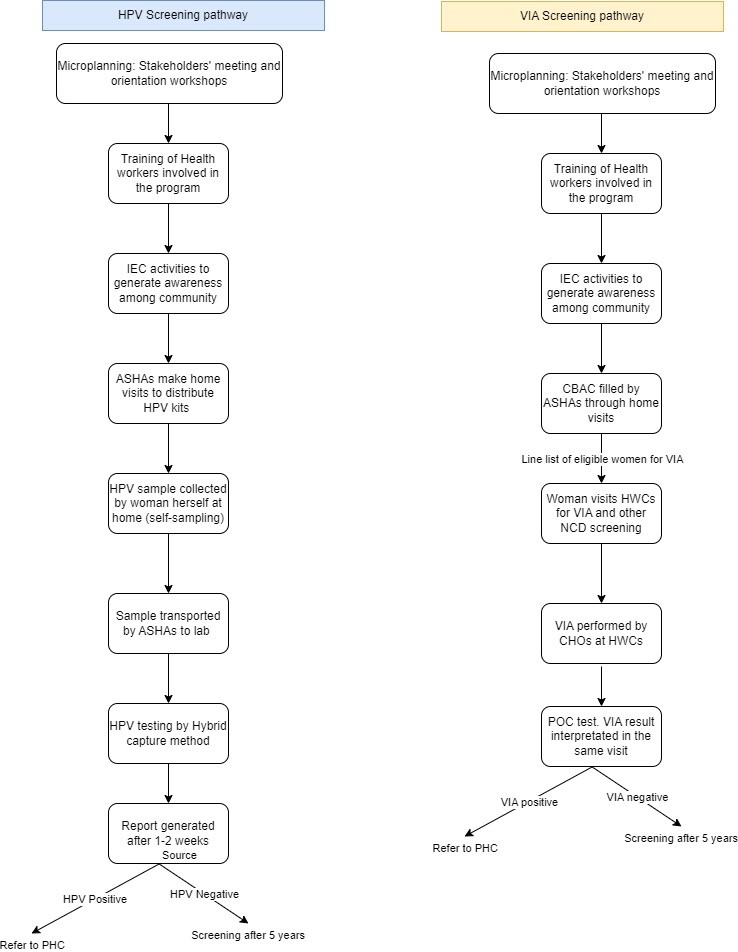

Supplement: S1 Fig — (DOCX) [file pone.0300556.s003.docx]
